# Transcriptional regulatory programs underlying barley germination and regulatory functions of Gibberellin and abscisic acid

**DOI:** 10.1186/1471-2229-11-105

**Published:** 2011-06-13

**Authors:** Yong-Qiang An, Li Lin

**Affiliations:** 1 US Department of Agriculture, Agriculture Research Service, Midwest Area, Plant Genetics Research at Donald Danforth Plant Sciences Center; 975 N Warson Road, St. Louis, MO 63132, USA; 2 221 Morrill Science Center III, Department of Biology University of Massachusetts, 611 N. Pleasant St., Amherst, MA 01003, USA

## Abstract

**Background:**

Seed germination is a complex multi-stage developmental process, and mainly accomplished through concerted activities of many gene products and biological pathways that are often subjected to strict developmental regulation. Gibberellins (GA) and abscisic acid (ABA) are two key phytohormones regulating seed germination and seedling growth. However, transcriptional regulatory networks underlying seed germination and its associated biological pathways are largely unknown.

**Results:**

The studies examined transcriptomes of barley representing six distinct and well characterized germination stages and revealed that the transcriptional regulatory program underlying barley germination was composed of early, late, and post-germination phases. Each phase was accompanied with transcriptional up-regulation of distinct biological pathways. Cell wall synthesis and regulatory components including transcription factors, signaling and post-translational modification components were specifically and transiently up-regulated in early germination phase while histone families and many metabolic pathways were up-regulated in late germination phase. Photosynthesis and seed reserve mobilization pathways were up-regulated in post-germination phase. However, stress related pathways and seed storage proteins were suppressed through the entire course of germination. A set of genes were transiently up-regulated within three hours of imbibition, and might play roles in initiating biological pathways involved in seed germination. However, highly abundant transcripts in dry barley and *Arabidopsis *seeds were significantly conserved. Comparison with transcriptomes of barley aleurone in response to GA and ABA identified three sets of germination responsive genes that were regulated coordinately by GA, antagonistically by ABA, and coordinately by GA but antagonistically by ABA. Major CHO metabolism, cell wall degradation and protein degradation pathways were up-regulated by both GA and seed germination. Those genes and metabolic pathways are likely to be important parts of transcriptional regulatory networks underlying GA and ABA regulation of seed germination and seedling growth.

**Conclusions:**

The studies developed a model depicting transcriptional regulatory programs underlying barley germination and GA and ABA regulation of germination at gene, pathway and systems levels, and established a standard transcriptome reference for further integration with various -omics and biological data to illustrate biological networks underlying seed germination. The studies also generated a great amount of systems biological evidence for previously proposed hypotheses, and developed a number of new hypotheses on transcriptional regulation of seed germination for further experimental validation.

## Background

Seed germination is a complex multi-stage developmental process important to plant development, plant evolution, and agricultural production. Strictly defined, germination begins with the uptake of water by dry quiescent seeds and ends with the visible emergence of an embryo tissue from its surrounding tissues. However, in many scientific literatures and agronomic research, seed germination often broadly includes early seedling growth, a process which ends with the start of autotrophic growth or the emergence of seedling from soil [1]. Seed germination is accompanied with many distinct metabolic, cellular and physiological changes. For example, upon imbibition, the dry quiescent seeds take up water and rapidly resume many fundamental metabolic activities such as respiration, RNA and protein synthesis machinery, as well many enzyme activities using surviving structures and components in the desiccated cells. Meanwhile, dry seeds gradually lose stress tolerances, such as desiccation tolerance, over the course of seed germination. These combined biological activities transform a dehydrated and resting embryo with an almost undetectable metabolism into one with vigorous metabolism calumniating in growth [2,3].

GA and ABA are two key phytohormones regulating seed germination and seedling growth. It is believed that GA and ABA play antagonistic roles in regulating seed germination and their ratios govern the maturation versus germination pathways that embryos will take after they complete rudimentary organogenesis [4,5]. It was proposed that GA enhances seed germination and seedling growth. Maturing maize embryos require GA for germination in culture. Treating maize embryos with GA synthesis inhibitors also decrease both the rate of germination and the fraction of embryos that germinate [4]. Treatments that promote *Arabidopsis *germination, such as cold and light, are often correlated with an increase in endogenous GA [6]. It has been showed that GA-deficient *Arabidopsis *and tomato mutants are impaired in seed germination [7,8]. It is proposed that a conserved DELLA protein negatively mediates GA regulation of seed germination and seedling growth [9-13]. However, the biological networks underlying GA regulation of seed germination and seedling growth are largely unknown. In germinating cereal grains, GA is primarily synthesized in the embryo and is then relocated to aleurone tissues where it induces synthesis of hydrolytic enzymes. The hydrolytic enzymes are further secreted into starchy endosperm to mobilize seed storage reserve to provide nutrients and energy for embryo growth and differentiation before an autotrophic phase is fully established. It is believed that GA induction of hydrolytic activities mainly occurs in the post-germination phase to support seedling growth [14]. However, the requirement of GA in early barley germination remains to be determined. In contrast, ABA content increases dramatically in most plant species during seed maturation, and induces the production of seed storage and desiccation tolerant proteins to prepare the seeds for undergoing desiccation and to produce energy and nutrient reserve for later seed germination [15-18]. ABA also suppresses expression of many hydrolytic enzyme genes to prevent viviparous germination [19,20]. Recent evidence suggests that other phytohormones, including auxin and ethylene, play roles in regulating seed germination [21,22].

Barley germination and seedling growth have been investigated extensively due to its importance in barley agriculture and the brewing industry [23]. A wealthy amount of diverse biological data from barley germination has been accumulated [24]. In addition, barley aleurone from germinating barley grains has been established as a model system to study the mode of action on GA and ABA response pathways and their regulatory functions in barley seed germination [25]. Recently, Barley Genome GeneChips containing approximately 22,700 genes were used in examining the transcriptome of barley aleurone in response to GA, ABA, and the inactivation of SLN1 proteins. The analysis identified 1328 GA and 206 ABA responsive genes and revealed that transcriptomes of barley aleurone respond antagonistically to GA and ABA treatments [20]. Loss-of-function of the DELLA protein, SLN1, activates barley aleurone transcriptomic programs in response to GA [26]. A great number of studies examined transcriptomes of *Arabidopsis *germinating seeds and tissues to study seed germination in response to developmental regulation, genetic variation, and environmental signals at a system level [27-30]. Transcriptomes of various germinating tissues in barley have been determined using a variety of transcript profiling technologies [31-35]. Barrero et.al compared the transcriptomes of coleorhiza and roots from dormant and after-ripened barley embryos at 8 and 18 hours after imbibition and characterized the dormancy related transcriptomic changes. Screenvasulu et. al performed a transcriptome analysis of endosperm and embryo at barely grain maturation, desiccation, and early seedling growth stages, and revealed a smooth transition in the transcriptional program between late seed maturation and early seedling growth within embryo tissues [33]. However, the research mainly focuses on post-germination processes. No germinating barley tissues prior to emergence of coleorhiza from grains were examined. It has been well demonstrated that the activities of many germination related gene products and biological pathways are subject to strict developmental regulation. However, an in-depth and comprehensive transcriptomic characterization of germinating barley representing distinct and well defined developmental stages over the entire course of seed germination are not available. To fill the gap, the studies carefully examined several physiological and morphological characteristics of barley germination and seedling growth, and selected six distinct developmental stages that represent the entire process of barley germination from grain imbibition to early seedling growth for transcriptome analysis. Extensive bioinformatic analysis of the dynamic transcriptomic data delineates the transcriptional regulatory program underlying barley germination and seedling growth at gene, pathway and systems levels.

## Results and Discussion

### Distinct Physiological and Developmental Stages of Barley Germination

One of the experimental objectives is to determine dynamic changes in transcriptomes of barley over the course of seed germination, and to further illustrate the transcriptional regulatory program underlying barley germination and its associated biological pathways. However, expression of germination important genes and biological pathways is often subjected to strict developmental regulation over the course of seed germination. It is crucial to examine transcriptome of barley representing well defined and distinct physiological stages. Morphology, water up-take, amylase activity and loss of seed desiccation resistance are important characteristics of germinating barley [2]. Having examined these characteristics of germinating barley over the course of seed germination, six developmental stages representing distinct physiology of barley over the course of seed germination were selected and referred as S0 to S5 stages.

**Figure 1:**
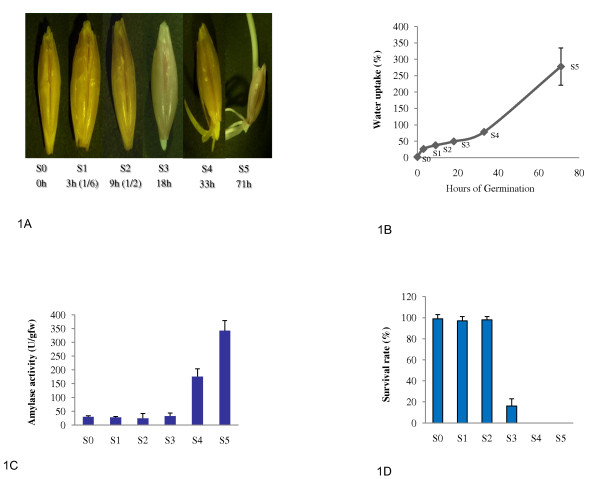
**Morphology and Physiology of Germinating Barley at Each Developmental Stage**. Figure 1A. Morphology and time points of germinating barley at each developmental stageThe morphology of germinating grains and seedlings at each developmental stage and the typical time taken for dry mature grains to reach each stage are shown. The relative times in reference to the time taken to reach S3 stage are indicated in the parenthesis. S3: Coleorhiza emerging from grains at 18 hours of germination. S4: the rootlet length is half that of its grain at 33 hours of germination. S5: the shoot is 3 times as long as its grain at 71 hours of germination. Figure 1B. Water content of germinating barley at each developmental stageThe fresh and dried weights of 10 germinating grains or seedlings at each developmental stages defined as in the Figure 1A were measured. The water content in germinating barley at each stage is indicated as Y axis as a percentage of the dry weight. The representative time point of germination at each stage is indicated as X axis. Standard derivations of the three replications are indicated as error bars. Stages are marked. Figure 1C. Alpha-amylase activity in germinating barley at each developmental stageThe X axis indicates the development stages. The average amount of maltose in umole produced per gram of fresh examined tissues (U/gfw) and the standard derivation of three replications are indicated on Y axis. Figure 1D. Desiccation resistance of germinating barley at each developmental stage The germinating barley at each developmental stage were dehydrated, and then re-germinated. The percentage of the dehydrated germinating barley that could revive to their growth was defined as survival rate and indicated as Y axis to measure desiccation resistance of the germinating barley. The X axis indicates developmental stages. Standard derivations of the three replications are indicated as error bars.

Figure 1A shows the morphology of germinating barley at each developmental stage and time typically taken for dry mature grains to reach the given stage. For example, S3 stage marked the end point of germination process, and can be easily identified by the visible coleorhiza emergence from the grains, which typically occurred at 18 hours of germination. No morphological changes were observed for the germinating grains prior to the S3 stage. Germinating grains at time points of 1/6 and 1/2 of the time typically taken for coleorhiza to emerge from germinating grains were referred to S1 and S2 stages to represent the early stages of germination. Figure 1B shows that water uptake of germinating barley had three phases over the course of seed germination as previously described [1]. The water content of germinating barley rapidly increased between S0 and S1 stages at a rate of 7.1% per hour. However, the water uptake slowed down dramatically after the S1 stage to the lowest rate of 1.2% per hour between S2 and S3 stages. Following the low-point, water uptake gradually increased to a higher rate of 5.2% per hour between stages S4 and S5 (Figure 1B). Alpha-amylase activity exemplifies the mobilization of starch storage reserves over the course of seed germination. While there was little change in alpha-amylase activity until the S3 stage, a dramatic activity increase occurred between stages S3 and S4, following the emergence of coleorhiza from germinating grains (Figure 1C). Mature dry seeds are highly resistant to desiccation and many other abiotic and biotic stresses. Over the process of grain germination, grains gradually lose desiccation resistance and those stress tolerances. No significant change in desiccation resistance was observed for the germinating grains until the S2 stage (Figure 1D). However, the survival rate of dehydrated grains at S3 stage dropped to 14.7% (Figure 1D). No germinating barley at S4 could be revived after dehydration. Barley grains completely lost their desiccation resistance over the period after grains finished their germination at S3 stage and before the production of amylase increases significantly at S4 stage. Thus, the six well characterized developmental stages defined above should cover the entire spectrum of physiological changes in barley from initial grain imbibition to early seedling growth. The germinating barley at each of the six stages should provide a representative and distinct physiological and developmental stage, and can be accurately and easily identified based on the relative timing of germination and morphology of seedlings.

It is a great challenge to accurately define and identify physiology and developmental stages of germinating seeds. Time points post imbibition are widely used to define the developmental stages of seed germination. However, seed germination rates are significantly affected by genotypes, physiology of dry seeds and germination environments. Individual dry seeds from the same harvest do not always germinate uniformly due to heterogeneity of seed maturity [2]. Although a large amount of diverse biological data and results have been reported in the previous germination studies, it faces a great difficulty to compare or integrate those data because the developmental stages of the germinating tissues used in most of those studies are not well defined. The relative timing of germination and the morphology of seedling described above could be used as an accurate and facile approach to identify germinating grains and seedlings equivalent to each of the six developmental stages and control variation of germination rates caused by those factors in other cereal species.

### A Transcriptomic Switch Correlated to the Morphological and Physiological Transition from Seed Germination to Seedling Growth

**Figure 2:**
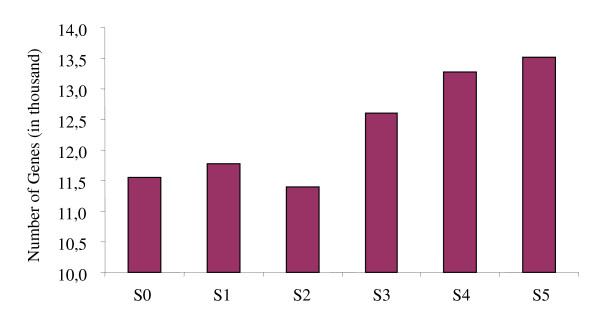
**Total Number of Detectable Transcript Species in Germinating Barley At Each Developmental Stage**. A probe set with present calls at P value cutoff of 0.05 in two of three replicates at each developmental stage is assigned as a transcript expressed at a detectable level. The number of probe-sets with "present" calls at each developmental stage is indicated as the Y-axis. The X-axis indicates the developmental stages.

Affymetrix Barley Genome GeneChip Arrays containing 22,792 probe sets [36] were used to examine the transcriptomes of germinating barley at the S0, S1, S2, S3, S4 and S5 stages. Three GeneChip assay replications each from an independent germination experiment were conducted for each developmental stage to control biological and technical variation. Figure 2 summarizes the number of mRNA species accumulating at a detectable level at each developmental stage. Over 50% of the 22,792 examined transcript species were accumulated at detectable levels in the dry grain. However, mRNA complexity increased over the course of germination except for a decrease from the S1 to S2 stages. The most dramatic increase in mRNA complexity occurred between the S2 and S3 stages. It is consistent with the previous reports that approximately 50% of examined transcripts are accumulated at a detectable level and encode all functional categories of proteins in dry seeds of divergent plant species [27,28,33]. Although a number of transcripts encoding seed maturation specific proteins such as seed storage proteins have been demonstrated to degrade over the course of germination [31], it is likely that many of them are preserved and continue to function through seed germination, at least through the early seed germination. It was shown that germination of *Arabidopsis *seeds can be blocked by a translational inhibitor, cycloheximide, but not by a RNA polymerase II inhibitor, alpha-amanitin [37,38]. Thus, it is likely that the potential of germination is largely programmed in the seed developmental process.

**Figure 3:**
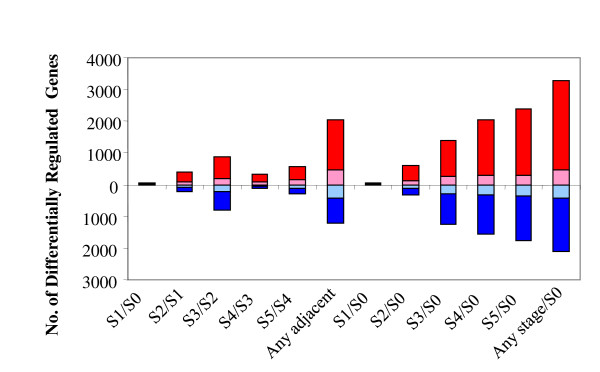
**Summary of Differentially Regulated Genes over the Course of Germination**. Differentially regulated genes were identified by either comparison of adjacent stages (left 6 bars) or comparing each stage to S0 stage (right 6 bars). The numbers of up- or down-regulated genes with statistical significance but less than three-fold change in their mRNA accumulation are indicated in pink or light blue. The numbers of up- or down-regulated genes with both statistical significance and more than a three-fold change in their mRNA accumulation are indicated in red or blue. The total number of genes differentially regulated between any adjacent stages and between any stage and S0 stage are marked as "Any adjacent" and "any stage/S0".

GC-RMA algorithm was used to convert probe level data to expression measurement in the microarray experiments [39]. One-way ANOVA analysis identified 6157 genes whose transcript accumulation changed significantly over the process of barley germination with a False Discovery Rate (FDR) of 0.05 (See Additional file 1). 5382 genes were differentially regulated between S0 stage and any other developmental stage. Of the 5382 genes, 4493 genes (84%) showed more than a three-fold change, indicating that most of the differentially regulated genes changed dramatically over the course of seed germination (See Figure 3 and See Additional file 2). Of the 4493 genes, 2,816 genes were up-regulated while 1,688 genes were down-regulated. There were 63% more up-regulated genes than down-regulated genes. This is consistent with increasing complexity of mRNA species and biological processes in germinating grains over the course of germination.

Comparison of any two adjacent developmental stages shows that 3267 genes changed significantly in their mRNA accumulation (Figure 3 and Additional file 2). 2390 of the 3267 genes had more than a three-fold mRNA accumulation changes. Interestingly, 1295 genes showed differential accumulation of their mRNAs within the 9 hours of germination between the S2 and S3 stages while only 310 genes were differentially expressed within following 15 hours between S3 and S4 stages. Both the biggest increase in mRNA complexity and the highest number of differentially regulated genes were observed between the S2 and S3 stages. Thus, a dramatic transcriptional program switch occurred between the two developmental stages and co-occurred with morphological emergence of coleorhiza, dramatic decrease in desiccation resistance, initiation of enzymatic alpha amylase activity increase, and the slowest water uptake over the course of germination. The transcriptional reprogram switch is likely to play a key regulatory role in transforming barley grains from germination to seedling growth.

The majority of differentially regulated genes between adjacent developmental stages showed more than three-fold changes in their transcript accumulations. To focus on the genes that are more likely to have functional significance in seed germination, the following analysis and description are only limited to the genes with more than three-fold changes unless specified otherwise.

### Conservation and Divergence of Highly Abundant Transcripts in Barley and *Arabidopsis *Dry Seeds

Table 1 lists top 100 barley probe-sets that have the highest signal intensity in barley dry grains. The probe-sets accounted for less than 1% of transcripts detectable in dry grains, and should represent highly abundant transcripts stored in the dry seeds. Those transcripts mainly encode proteins related to nutrient reservoir, stress tolerance, protein biosynthesis, glycolysis, lipid metabolism, oxidoreduction, and metal binding. A number of transcripts encoding proteins with unknown functions or related to other diverse functionalities were also found in the top 100 barley probe-sets.

**Table 1:** Comparison of Highly Abundant Seed Stored Transcripts in Barley and *Arabidopsis*

Barley Probe-Set ID	**Rank**^**1**^	Functional Category	Gene Annotation	**Rank**^**2**^	Ara ID	**Orthologs**^**3**^
Contig5481_at	32	nutrient reservoir	late embryogenesis abundant protein	70	AT3G53040	Y
HV09J08u_s_at	13	nutrient reservoir	cupin family protein	67	AT3G22640	Y
Contig1353_s_at	38	nutrient reservoir	cupin family protein	67	AT3G22640	Y
HD01C09w_s_at	63	nutrient reservoir	cupin family protein	67	AT3G22640	Y
Contig2408_at	52	nutrient reservoir	late embryogenesis abundant protein	151	AT3G15670	Y
Contig2407_s_at	59	nutrient reservoir	late embryogenesis abundant protein	151	AT3G15670	Y
Contig4008_at	83	nutrient reservoir	seed maturation protein PM28	146	AT3G12960	Y
Contig1832_x_at	2	nutrient reservoir	Late embryogenesis abundant protein	45	AT2G40170	Y
Contig1832_at	4	nutrient reservoir	Late embryogenesis abundant protein	45	AT2G40170	Y
Contig1830_at	6	nutrient reservoir	Late embryogenesis abundant protein	45	AT2G40170	Y
Contig1830_s_at	10	nutrient reservoir	Late embryogenesis abundant protein	45	AT2G40170	Y
Contig1832_s_at	14	nutrient reservoir	Late embryogenesis abundant protein	45	AT2G40170	Y
HVSMEi0008A06f2_s_at	55	nutrient reservoir	seed maturation protein PM41,	11	AT2G21820	Y
Contig4760_s_at	72	nutrient reservoir	late embryogenesis abundant protein	21	AT1G01470	Y
Contig811_x_at	7	nutrient reservoir	B3-hordein (clone pB7)			Y
EBed07_SQ001_B14_s_at	24	nutrient reservoir	seed storage protein			Y
Contig785_x_at	35	nutrient reservoir	hordein B precursor			Y
Contig523_x_at	82	nutrient reservoir	B3-hordein (clone pB7)			Y
Contig793_x_at	85	nutrient reservoir	B3-hordein			Y
HB01O23r_x_at	28	nutrient reservoir	hordein B precursor			N
Contig540_x_at	39	nutrient reservoir	B3-hordein (clone pB7)			N
Contig585_x_at	67	nutrient reservoir	hordein B precursor			N
Contig2519_x_at	27	Stress	chitinase			Y
Contig3288_x_at	56	Stress	heat shock protein 17.6-II	33	AT5G12030*	Y
Contig3286_s_at	91	Stress	17.6 kDa class II heat shock protein	33	AT5G12030*	Y
Contig2007_s_at	89	Stress	18 kDa class I heat shock protein	1	AT3G46230*	Y
Contig2010_at	97	Stress	17.4 kDa class I heat shock protein	1	AT3G46230	Y
Contig797_at	50	stress	thionin (THI2)	64	AT2G15010*	Y
HB18H23r_s_at	49	Stress	17.6 kDa class I small heat shock protein	404	AT1G53540	Y
HB16L13r_x_at	80	Stress	17.6 kDa class II heat shock protein			Y
Contig1713_s_at	22	stress	dehydrin (RAB18)	338	AT5G66400	Y
Contig1763_s_at	18	stress	PDF2.1; peptidase inhibitor	93	AT2G02120*	Y
HT11E22u_x_at	5	stress	gamma-thionin precursor			N
Contig375_s_at	16	stress	gamma-thionin precursor			N
Contig459_s_at	26	protein biosynthesis	elongation factor 1-alpha/EF-1-alpha	51	AT5G60390	Y
Contig1024_at	23	protein biosynthesis	40S ribosomal protein S8 (RPS8A)	368	AT5G20290	Y
EBed02_SQ003_C14_s_at	46	protein biosynthesis	40S ribosomal protein S8 (RPS8A)	368	AT5G20290	Y
HY09G23u_s_at	37	protein biosynthesis	elongation factor 1B alpha-subunit 2 (eEF1Balpha2)	470	AT5G19510	Y
HS09B02u_s_at	95	protein biosynthesis	eukaryotic translation initiation factor SUI1	377	AT4G27130*	Y
Contig2094_s_at	64	protein biosynthesis	40S ribosomal protein S23 (RPS23B)	474	AT3G09680*	Y
HM01F24T_s_at	92	protein biosynthesis	60S ribosomal protein L23 (RPL23C)	361	AT3G04400	Y
Contig545_s_at	88	protein biosynthesis	60S ribosomal protein L8 (RPL8A)	385	AT2G18020	Y
Contig692_s_at	94	protein biosynthesis	60S ribosomal protein L8 (RPL8A)	385	AT2G18020	Y
Contig1607_at	77	protein biosynthesis	eukaryotic translation initiation factor 5A,	313	AT1G26630	Y
rbaal18i13_s_at	9	protein biosynthesis	60S ribosomal protein L5			Y
HS18F06u_s_at	34	protein biosynthesis	60S ribosomal protein L7A (RPL7aB)			Y
HT06A08u_s_at	36	protein biosynthesis	60S ribosomal protein L10 (RPL10B)			Y
HA12A08u_s_at	44	protein biosynthesis	40S ribosomal protein S18 (RPS18C)			Y
Contig1809_at	48	protein biosynthesis	60S acidic ribosomal protein P2 (RPP2A)			Y
HW02F22u_s_at	54	protein biosynthesis	60S ribosomal protein L15 (RPL15B)			Y
Contig2290_s_at	65	protein biosynthesis	60S ribosomal protein L31 (RPL31C)			Y
Contig3535_s_at	71	protein biosynthesis	60S acidic ribosomal protein P3 (RPP3A)			Y
Contig1938_s_at	73	protein biosynthesis	60S ribosomal protein L15 (RPL15B)			Y
Contig1476_at	87	protein biosynthesis	60S ribosomal protein L21 (RPL21C)			Y
Contig2373_s_at	100	protein biosynthesis	60S ribosomal protein L24 (RPL24B)			Y
Contig726_x_at	68	protein biosynthesis	60S ribosomal protein L41 (RPL41D)			N
Contig107_s_at	96	protein biosynthesis	60S ribosomal protein L41 (RPL41D)			N
HU02F20u_s_at	11	protein degradation	ubiquitin-conjugating enzyme	132	AT1G64230	Y
Contig2088_s_at	81	protein degradation	TIBHB trypsin inhibitor			N
HT06G21u_s_at	40	glycolysis	enolase			Y
Contig940_s_at	74	glycolysis	fructose-bisphosphate aldolase, putative			Y
Contig1188_s_at	1	lipid metabolism	lipid transfer protein 6 (LTP6)	242	AT2G38530*	Y
HVSMEk0006G04r2_s_at	17	lipid metabolism	glycine-rich protein/oleosin	129	AT5G40420	Y
Contig3234_s_at	8	lipid metabolism	glycine-rich protein/oleosin	159	AT4G25140	Y
EBma08_SQ004_C15_s_at	99	metal binding protein	selenium-binding protein, putative			Y
Contig1432_at	12	metal binding protein	plant EC metallothionein-like family 15 protein	192	AT2G23240	Y
Contig2483_at	3	redox	oxidoreductase	148	AT1G54870	Y
Contig3461_at	86	redox	glutaredoxin, putative	171	AT5G63030*	Y
Contig2853_at	45	redox	antioxidant/thioredoxin peroxidase	359	AT1G48130	Y
HT11A05u_s_at	93	other	aldose reductase, putative	13	AT5G01670	Y
Contig5448_at	30	other	lactoylglutathione lyase family protein			Y
HY02N18u_s_at	33	other	nucleoside diphosphate kinase 1			Y
HM02P13u_s_at	70	other	S-adenosylmethionine synthetase 2			Y
Contig146_s_at	20	other	glycine-rich RNA-binding protein			Y
Contig97_at	41	other	TCTP (TRANSLATIONALLY CONTROLLED TUMOR PROTEIN)	113	AT3G16640*	Y
Contig97_s_at	31	other	translationally controlled tumor family protein	113	AT3G16640	Y
HT03K14r_s_at	66	other	tonoplast intrinsic protein, alpha/alpha-TIP (TIP3)	63	AT1G73190	Y
Contig3690_s_at	53	other	AWPM-19-like membrane family protein	141	AT1G04560	Y
Contig1071_s_at	25	other	glycine-rich protein			Y
HVSMEi0013L12r2_s_at	29	other	plastocyanin-like domain-containing protein			Y
Contig4431_s_at	84	other	F5 protein-related/4F5 protein-related			Y
Contig360_x_at	57	other	glycine-rich RNA-binding protein (GRP7)			Y
Contig4493_s_at	42	unknown	unknown			Y
Contig1955_s_at	58	unknown	unknown			Y
Contig1752_s_at	60	unknown	unknown			Y
HVSMEf0021D08f_s_at	61	unknown	unknown			Y
HK06G13r_s_at	78	unknown	unknown			Y
HS17I13u_s_at	98	unknown	unknown			Y
Contig1751_s_at	15	unknown	unknown			N
Contig11968_at	19	unknown	unknown			N
HB07K19r_x_at	21	unknown	unknown			N
Contig15682_at	43	unknown	unknown			N
Contig372_s_at	47	unknown	unknown			N
HD11C22r_s_at	51	unknown	unknown			N
HU03F22u_at	62	unknown	unknown			N
HB18O02r_at	69	unknown	unknown			N
EBpi07_SQ001_P12_at	75	unknown	unknown			N
HVSMEl0014O24f_x_at	76	unknown	unknown			N
Contig18451_at	79	unknown	unknown			N
Contig9754_at	90	unknown	unknown			N

1: rank of barley transcript abundance in dry seeds.

2: rank of *Arabidopsis *transcript abundance in dry seeds

3. Presence of *Arabidopsis *genes orthologous to the barley genes: Yes (Y) and No (N)

*: *Arabidopsis *genes homologous to the barley genes at e-value less than -10.

Transcriptomes of *Arabidopsis *dry seeds have also been extensively characterized [28,29,38]. Nakabayashi et. al identified 484 highly abundant transcripts in non-dormant *Arabidopsis *dry seeds. Comparing the two sets of *Arabidopsis *and barley genes identified 35 pairs of putative barley-*Arabidopsis *orthologs that are highly abundant in both barley and *Arabidopsis *dry seeds. In addition, ten pairs of homologous barley-*Arabidopsis *genes with e-value less than -10 have been identified in those functional categories. Those transcripts were found in all functional groups of highly abundant barley transcripts except for the groups of unknown functions and glycolysis pathways. Many of their encoded proteins and pathways have been previously reported to be highly accumulated in the dry seeds, and suggested to be involved in seed maturation and germination [40]. For example, increasing evidence indicates that germination of seeds is accompanied by extensive changes in the redox state of proteins [41,42]. Translation of dry seed stored transcripts is required for seed germination [37,38]. Monocot-dicot divergence occurred approximately 200 million years ago [43]. Gene expression patterns change quickly if they have no functional constrains [44-47]. Preserving high accumulation of those ancient gene transcripts and pathway transcripts in both barley and *Arabidopsis *dry seeds from their ancestor after 200 million years of independent evolution strongly suggests that those transcripts and pathways are functionally important to germination, and may contribute to the biological characteristics of germination shared by barley and *Arabidopsis*. Although barley and *Arabidopsis *have evolved as two distinct types of starchy and oil seed plants respectively over the 200 million years, it is likely that transcriptional programs and molecular mechanism underlying seed germination are highly conserved, particularly in biological pathways such as stress tolerance, nutrient reservoir and protein translation. Interestingly, two pairs of oleosin orthologous transcripts are highly accumulated in not only *Arabidopsis *seeds but also barley seeds. Oleosin is a highly accumulated protein in oil bodies that mainly stores triacylglycerol (TAG) as major reserve in mature seeds to provide energy for seed germination and seedling growth [48]. It is believed that oleosin plays important regulatory roles in oil body stabilization and size [49,50]. It was observed that oleosin proteins are highly abundant in oil bodies from *Arabidopsis *and *Brassica *seeds [51,52]. Oil bodies and expression of oleosin have also been observed in barley embryo and aleurone tissues [53]. Although barley and Arabidopsis evolve to use starch and oil as major storage reserve respectively to support seed germination and seedling growth, it seems that barley still preserve high accumulation of oleosin in seeds. It will be interesting in understanding their biological funcitons.

A significant number of the highly abundant barley seed transcripts have no orthologous genes in *Arabidopsis*; or their orthologs or strong homologs do not highly accumulate in *Arabidopsis *dry seeds. Some of the barley transcripts encode hordein proteins in nutrient reservoir, glycolysis pathway enzymes, proteins with unknown functions, and a number of proteins with other functions. Interestingly, fructose-bisphosphate aldolase and enolase transcripts in the glycolysis pathways are highly accumulated in the barley grains, but none of their *Arabidopsis *orthologs and strong homologs highly accumulates in *Arabidopsis *dry seeds. Thus, specific high accumulation of the glycolysis enzyme transcripts in starch barley dry grains suggest that barley has evolved an unique regulatory pathway to quickly activate glycolysis upon imbibition to support early energy-demanding biological process. It raises possibilities that those barley genes and/or their high accumulation patterns in dry grains have diverged from their *Arabidopsis *orthologs after monocot-dicot occurred, and contribute to characteristics of barley seeds distinct from that of *Arabidopsis*. The comparative studies on the highly abundant barley and *Arabidopsis *transcripts should provide insight into molecular mechanism underlying conserved and divergent characteristics of barley and *Arabidopsis *germination.

### The Early and Transient Regulation of Barley Germination

**Figure 4:**
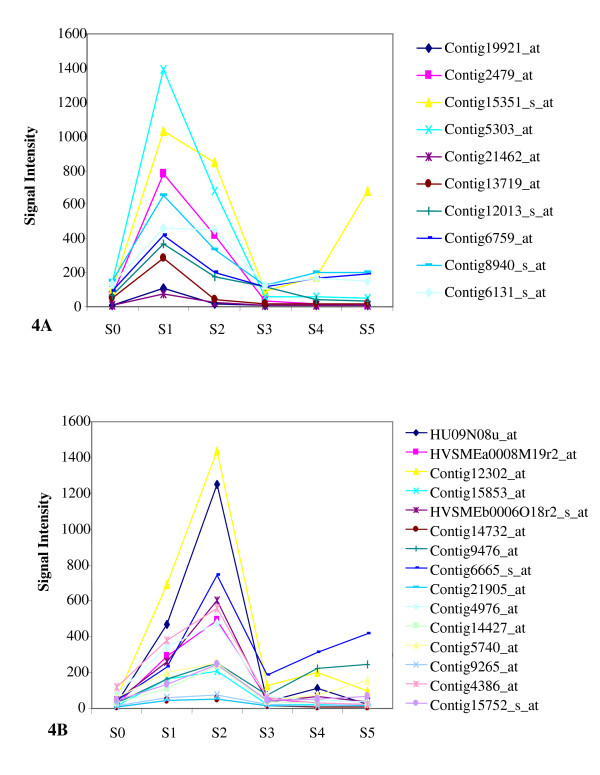
**Expression Patterns of the Genes Up-Regulated in The first Three hours of Germination**. The expression pattern of each gene with peak expression at S1 stage is shown in 4A and those with peak expression at S2 are shown in 4B. The signal intensity of each gene is expressed on the Y-axis. The probe-set ID for each gene is shown.

Transcriptional changes occurred as early as in the first three hours of germination. Forty -seven genes were differentially regulated between S0 and S1 stages. Twenty-five of these genes had more than 3 fold increases in their mRNA accumulation. Ten of the 25 up-regulated genes reached the highest expression level at S1 stage (Figure 4A), and then gradually dropped to the levels of mature grains at S3 stage. This group of genes encoded two zinc finger proteins, one Avr9/Cf-9 rapidly elicited protein, one DRE-binding protein, one arabinogalactan-like protein, two glutaredoxin and three proteins with unknown functions. The accumulation of the other 15 gene transcripts increased at S1 stage and reached the maximum level at S2 stage (Figure 4B). Those genes encoded WRKY family transcription factors, DnaJ-like proteins, an Avr9/Cf-9 rapidly elicited protein, a beta-glucan elicitor receptor, AAA-type ATPase, serine/threonine phosphatase 2C, ARM repeat protein, oxysterol-binding protein-like protein and proteins with unknown functions. Interestingly, the transcript accumulation for the majority of these genes also dropped to the levels of mature seeds at S3 stage. Many of the early induced genes encoded transcription factors and receptor proteins. Early differential expression of genes in response to GA has been successfully used as a criterion to identify regulatory genes in the GA response pathway in *Arabidopsis *[54]. It was shown that transcriptional changes can be detected in 15 minute of imbibition in *Arabidopsis*. However, much lower number of genes are up-regulated than down-regulated in *Arabidopsis *seeds within the first hour of imbibition. Only four transcripts are up-regulated while eighty-three transcripts are down-regulated within the first hour of imbibition [29]. Such a transient and early induction accumulation pattern was also observed in the rice germination. A cluster of rice transcripts are up-regulated at early stage of germination, and reach its peak in their abundance at 1 or 3 hours after imbibition, and then decreased to low levels again at 12 hours after imbibition [55]. The early and transient induction of those genes during seed germination raises a possibility that the genes could potentially play regulatory roles in initiating transcriptional regulatory cascades and signaling transduction pathways underlying barley germination. Some of the genes encode transcription factors and regulatory components in signaling pathways that are potentially related to seed germination. For examples, the probe sets, Contig 9265_at, encodes a serine/threonine phosphatase type 2C (PP2C), and was up-regulated by 3.5 and 4 folds at S1 and S2 stages respectively. It has been shown that serine/threonine protein phosphatase 2Cs (PP2C) can suppress ABA signaling pathways in *Arabidopsis *[56]. The loss-of-function of *Arabidopsis *ABI1 and ABI2 that encode protein phosphatase 2Cs increases seed dormancy and enhances responsiveness to ABA [57]. In addition, it was reported that two rice WRKY genes could repress ABA induction of the HVA22 promoter [58]. It is well documented that ABA promotes seed dormancy and inhibits seed germination and seedling growth [59,60]. The early and transient transcriptional up-regulation of negative regulators in ABA signaling pathways suggests that the induced accumulation of the mRNA species might suppress ABA function at the early stage of seed germination to promote seed germination.

### Three Distinct Phases of Transcriptional Regulatory Program Underlying Barley Germination

**Figure 5:**
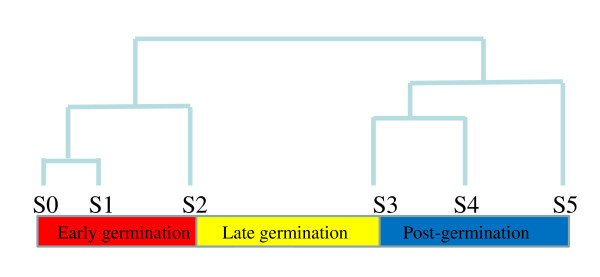
**Three Phases of the Transcriptional Regulatory Program Underlying Barley Germination**. The normalized mRNA intensities of the 6157 differentially regulated genes at the six developmental stages were subject to hierarchical cluster analysis. The six developmental stages were clustered into two groups with a threshold distance of 174. The transcriptional regulatory program underlying barley germination process was divided into three germination phases, early, late and post-germination phases.

Hierarchical clustering of all examined stages based on the normalized mRNA accumulation of the 6157 differentially regulated genes revealed that the six developmental stages were further clustered into two groups with the threshold distance of 174 (Figure 5). The developmental stages of S0, S1, and S2 were clustered into one group while S3, S4 and S5 were grouped into another. Although S2 and S3 stages were developmentally adjacent to each other with only 9 hours of interval, they were clustered in two separate groups, which is consistent to the dramatic increase in mRNA complexity and the highest number of differentially regulated genes observed between the two adjacent developmental stages. The clustering data further supports that a dramatic transcriptional program switch occurred between the two developmental stages. It also revealed that the transcriptional regulatory program underlying germination was composed of three distinct transcriptional phases of germination. The three distinct phases of the transcriptional regulatory program are well correlated to the three phases of water up-takes of seed germination, and are referred to early (from S0 to S2), late (S2 to S3) and post- (S3 to S5) germination phases.

**Figure 6:**
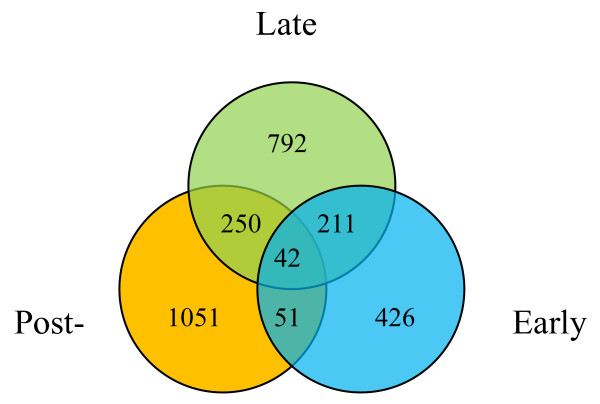
**Venn Diagram of the Early, Late, and Post-germination Regulated Genes**. The genes differentially regulated by early (blue), late (green), and post- (orange) germination phases were compared and displayed as a Venn diagram. The numbers of differentially regulated genes specific to one, two or all processes are indicated in the Venn diagram.

A total of 730, 1295, and 1394 genes changed significantly for more than three folds in their transcript accumulation during early, late and post-germination phases respectively (Additional file 3). Those genes were named as early germination, late germination and post-germination regulated genes. Figure 6 shows the Venn diagram of the early, late and post-germination genes with 426 genes specifically and differentially regulated during the early germination phase, 792 genes during the late germination phase, and 1051 genes during the post-germination phase (See Additional file 3). It is likely that those genes are responsible for the biological changes specifically occurring in their corresponding germination phase. 42 genes were differentially regulated by all three developmental phases (Additional file 3), while 51 genes by both early and post-germination phases, 250 by late and post-germination phases and 211 genes by early and late germination phases.

### Biological Pathways Differentially Regulated in Each Germination Phase

**Figure 7:**
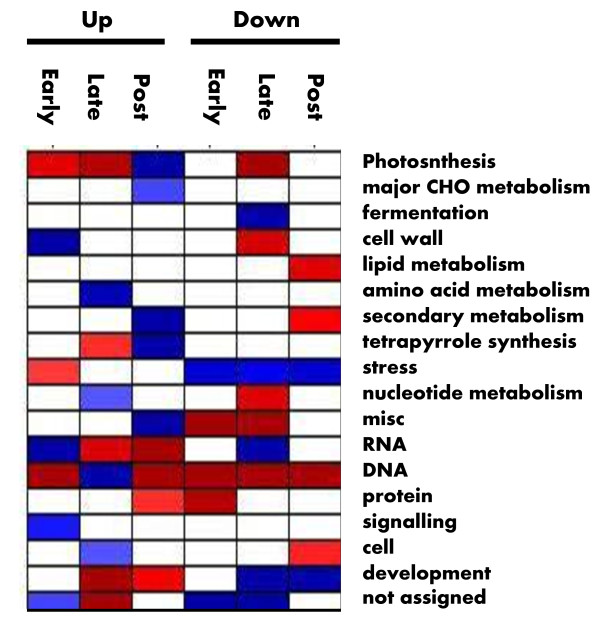
**Over-Representation of Genes Regulated Differentially by Each Germination Phase in Master Bins**. The representation analysis was conducted for the genes differentially regulated by each of the three germination phases. Log3 fold change values and a Hvu_Affy.m02 mapping file was used in the PageMan analysis. Fisher's exact test and an ORA Cutoff value of 1 were used. A false color scale of 3 was used to indicate the statistic Z value. Blue indicates significance in over-representation while red denotes significance in under-representation. Over- and under-represented master bins are annotated with functionalities on the left and the germination phase and regulation patterns on the top.

**Figure 8:**
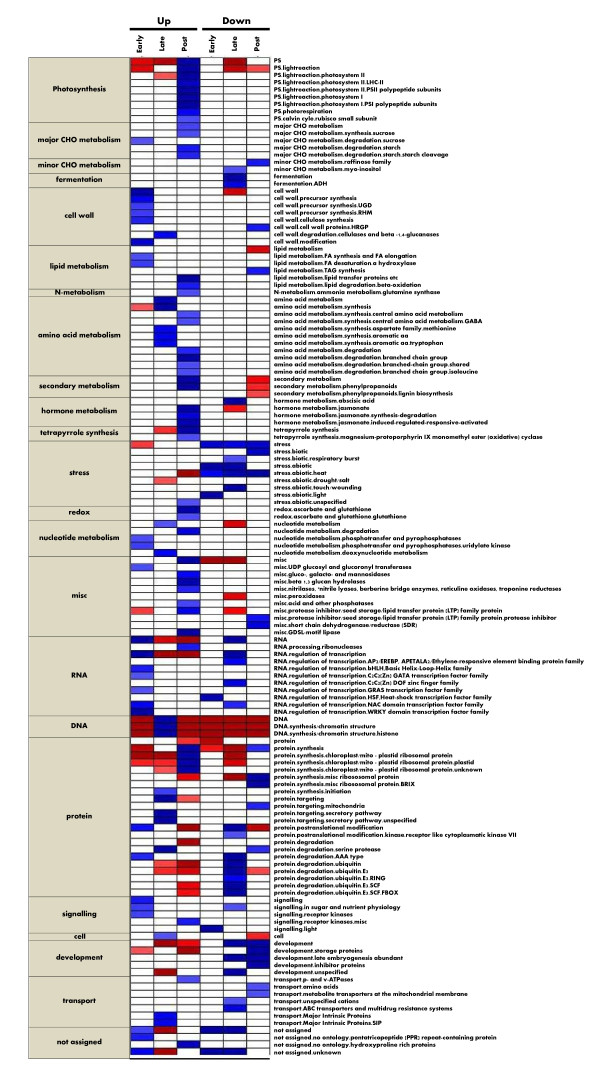
**Over-Representation of Genes Regulated Differentially by Each Germination Phase in All Functional Bins**. The representation analysis was conducted for the genes differentially regulated by each of the three germination phases. Log3 fold change values and a Hvu_Affy.m02 mapping file was used in the PageMan analysis. Fisher's exact test and an ORA Cutoff value of 1 were used. A false color scale of 3 was used to indicate the statistic Z value. Blue indicates significance in over-representation while red denotes significance in under-representation. Over- and under-represented bins are annotated with functionalities on the right and the germination phase and regulation patterns on the top.

Identification of a large number of differentially regulated genes in each germination phase enables the studies to identify biological pathways and functional groups that are transcriptionally up-regulated or down-regulated at a systems level using MapMan and PageMan tools. In the analysis, the probe-sets on Barley Genome GeneChip were assigned into 35 major functional bins based on their molecular functionalities such as metabolic pathways, signaling pathways and gene families. Each bin is further divided into sub-functional bins [33]. PageMan and MapMan software tools were used to determine the statistic probability of over- or under-representation of the early, late or post- germination regulated genes in each bin and sub-bin [61,62]. The representation analysis revealed that 18 bins and 138 sub-functional bins were up-regulated or down-regulated by at the least one of the three germination phases with over-representation Z-value of more than 1 (Figure 7 and 8). Having only considered the pathways with statistical significance, the systems and over-representation analysis offers a more effective approach to discover biological pathways/processes that are transcriptionally regulated during seed germination and potentially important to seed germination.

#### Up-regulation of Regulatory Components and Cell Wall Metabolism in Early Germination Phase

The representation analysis of the 730 early germination regulated genes revealed that bin and sub-bins with regulatory and signaling functionalities were preferentially up-regulated in the early germination phase (Figure 7 and 8). The early germination up-regulated genes were over-represented in the bins of Cell Wall, RNA and Signaling. Although the RNA bin is composed of three sub-functional bins, RNA Processing, RNA Transcription, and Regulation of Transcription [61], only the Regulation of Transcription sub-bin was preferentially up-regulated in the early germination phase. All up-regulated sub-bins in Regulation of Transcription were transcription factor families including Helix-Loop-Helix, C2C2(Zn)GATA, GRAS, NAC domain, and WRKY transcription factor families. In the Signaling bin, the sub-functional bins of Signaling in Sugar and Nutrient Physiology and Receptor Kinases were up-regulated. Sub-functional bin of Protein Post-Translational Modification, which often plays regulatory functions in activities of proteins, was also preferentially up-regulated in early germination phase. The regulatory and signaling functional categories were specifically and transiently up-regulated in the early germination phase. In contrast, the late or post-germination up-regulated genes were under-represented in Regulation of Transcription category while late germination down-regulated genes were over-represented in some regulatory and signaling functional categories. The specifically and transiently up-regulation of the regulatory and signaling functionalities in the early germination phase strongly suggested that a major molecular event in early germination phase is to transcriptionally induce genes encoding regulatory and signaling components, and therefore to initiate a variety of transcriptional regulatory cascades and signaling pathways involved in germination and seedling growth.

The bin of Cell Wall and its sub-functional bins of Cell Wall Precursor Synthesis, Cellulose Synthesis and Cell Wall Modification were also up-regulated in the early germination phase. A total of 30 cell wall related genes were differentially regulated during early germination; and twenty-seven of them were up-regulated (See Additional file 4). The biased up-regulation of cell wall metabolism is consistent with the proposed roles of cell wall synthesis, degradation and modification in seed germination. Endosperm weakening is considered a major feature associated with endosperm rupture by the expanding radicle, a major regulation point for germination potential [59,63]. In several species endosperm weakening has been associated with the induction of cell wall remodeling enzymes. They include endo-beta mannanase, beta-1,3-glucanases, expansins, xyloglucan endotransglycosylase, pectin methylesterase, ploygalacturonase and arabinogalactans [59]. Transcripts encoding all of the enzymes except endo-beta mannanase were observed in the set of the early germination up-regulated transcripts. Barrero, et.al showed that a number of cell wall degradation related genes are preferentially expressed in after-ripening barley coleorhiza for both 8 and 18 hours of hydration, and are proposed to be associated with breaking seed dormancy [34]. Interestingly, the *Arabidopsis *ortholog of endo-beta mannanase was also down-regulated at three hours after imbibition. Fourteen of the barley cell wall related genes have *Arabidopsis *orthologous genes that are differentially regulated by more than two folds. All of those fourteen pairs of orthologs were up-regulated in response to early germination in both barley and *Arabidopsis*, strongly supporting that those cell wall genes have functional significance in seed germination. It was shown that a GATA zinc finger transcription factor functions as a positive regulator of germination, and is required to facilitate endosperm rupture in *Arabidopsis *[64]. Interestingly, three probe-sets (Contig3743_at, 17684_at and 4186_at) were annotated as GATA zinc finger transcription factors and up-regulated by more than five folds within the early phase of barley germination. Thus, it is likely that early germination process turns on the transcriptional regulatory pathway underlying cell wall metabolism activity to weaken coleorhiza and facilitate root emergence.

The early germination down-regulated genes were over-represented in the Stress bin. In this bin, only the sub-functional bins of the Heat and Light stresses in Abiotic Stress were preferentially down-regulated. Interestingly, the sub- bins of Light Signaling and Heat-shock Transcription Factors were also the only two sub-functional bins of Signaling and Regulation of Transcription over-represented in early germination down-regulated genes. The coordinated down-regulation of both heat and light-stress pathways and their corresponding transcription factor and signaling genes in early germination phase suggests that biological networks underlying heat- and light-stress response were suppressed in early germination phase and that down-regulation of those transcription factors and signaling component genes are likely to lead to suppression of the biological networks.

No significant changes in the morphology, desiccation resistance, or amylase activity other than water uptake were observed in germinating grains within the first nine hours of the early germination phase. However, transcript accumulation of 730 genes changed significantly during early germination phase; and germinating grains already activated their transcriptional machinery and reprogrammed their transcriptional expression to synthesize and degrade a specific set of transcripts. Interestingly, the studies did not observe that genes encoding mRNA synthesis and degradation machinery proteins were preferentially up-regulated in the early germination phase. It raises the possibility that the germinating grains utilize RNA synthesis and degradation machinery preserved in the mature seeds to support the RNA metabolism in the early germination phase.

#### Up-regulation of Metabolic Pathways and Chromatin Structure in Late-germination Phase

Over the following nine-hour late germination phase from S3 to S4 stages, dramatic morphological and physiological changes occurred. Coleorhiza emerged from germinating grains; and desiccation resistance decreased significantly. However, water-uptake rate reached its lowest level. A total of 1295 genes were differentially regulated over the late germination process. The bins of Amino Acid Metabolism, Nucleotide Metabolism, Cell and DNA were preferentially up-regulated in the process. In Amino Acid Metabolism, the Amino Acid Synthesis pathways, including Methionine and Aromatic Amino Acid Synthesis pathways, but not the Amino Acid Degradation pathways, were preferentially up-regulated. Serine Protease in the protein degradation pathway, Protein Synthesis Initiation, Major Intrinsic Protein Transport and Secretory Pathways in Protein Targeting, and Cell Wall Degradation were preferentially up-regulated in late germination process. In addition, the bin of Cell, which includes genes related to cell division, cell cycle and vesicle transport, were preferentially up-regulated in the late germination phase.

It is striking that the sub-functional bins of Chromatin Structure and Histone family were over-represented in late germination up-regulated genes with a very high Z value of greater than 18 (See Table 2). A total of 126 genes encoded histone proteins H2A, H2B, H3 and H4. All of the histone genes were up-regulated in the late germination phase. In contrast, the functional categories of Chromatin Structure and Histone were under-represented in both up-regulated and down-regulated genes in early and post-germination phases as well as in down-regulated genes in late germination phase. Thus, histone genes and chromatin structure related genes were specifically and preferentially up-regulated in the late germination phase. Histone modification and chromatin remodeling play important roles in reprogramming transcriptional programs, and have been evidenced to play regulatory roles in the seed dormancy and germination [65-67]. It was shown that the mutation of histone monoubiquitination genes in *Arabidopsis *reduced ubiquitinated forms of histone H2B, and altered expression levels for several dormancy-related genes [66]. A transient histone deacetylation event occurs during seed germination one day after imbibition, and serves as a key developmental signal that affects the repression of a number of histone deacetylase regulated genes [67]. Gene expression patterns changed very quickly if there is no functional constrains after gene duplication [68]. The extremely biased up-regulation of so many histone genes provide strong evolutionary evidence that they might play roles in chromatin remodeling and reprogramming the transcriptional regulatory program for seeds to switch from germination to post-germination seedling growth. Thus, over-representation analysis data suggests that germinating grains activate many metabolic pathways in late germination phase to produce amino acid and nucleotides, establish and maintain protein synthesis and transporting machinery, and remodel chromatin structure to support the cell division and expansion occurring in late and early post-germinations.

**Table 2:** Summary of Differentially Regulated Genes in the Bin of DNA

	GeneChip	Early germination			Late germination			Post-germination		
Binname	Total	Regulated	Up-	Down-	Regulated	Up-	Down-	Regulated	Up-	Down-
DNA	469	10	10	0	143	141	2	9	4	5
DNA.synthesis/chromatin structure	379	8	8	0	142	141	1	6	2	4
DNA.synthesis/chromatin structure.histone	217	7	7	0	126	126	0	2	1	1
DNA.repair	26	0	0	0	0	0	0	2	1	1
DNA.unspecified	64	2	2	0	1	0	1	1	1	0

The late germination down-regulated genes were over-represented in the functional bins of Fermentation, Stress, RNA and Development (Figure 7). Some of the sub-functional bins, such as abiotic stress and LEA, were down-regulated in both late and post-germination phases. Many of the down-regulated functionalities such as LEA, Abiotic Stress and ABA Metabolism are highly expressed during seed maturation and involved in seed maturation and stress tolerance such as desiccation tolerance in mature grains [33,59,69]. Down-regulation of those functionalities also co-occurred with loss of desiccation resistance in the late germination period, and is likely to contribute to loss of desiccation tolerance during seed germination.

#### Up-regulation of Photosynthesis, Degradation, Secondary Metabolic Pathways in Post-germination Phase

Post-germination phase represented the fifty-three hours of seedling growth from the S3 to S5 stages. The bins of Photosynthesis, Major CHO Metabolism, Secondary Metabolism, Tetrapyrrole Synthesis were preferentially up-regulated while functional categories of Stress and Development were down-regulated (Figure 7).

**Figure 9:**
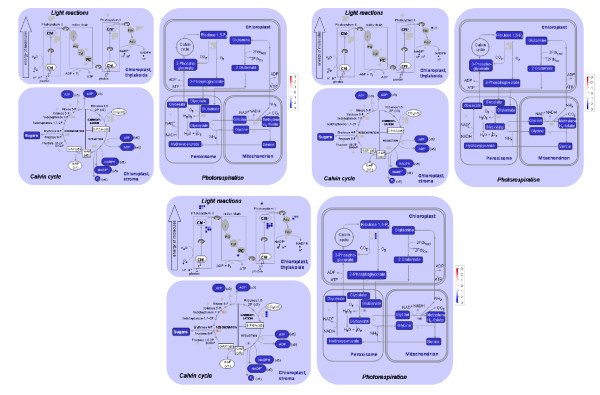
**Differentially Regulated Genes in the Photosynthesis Pathways over Each of Three Germination Phases**. The Light Reactions, Calvin Cycle and Photorespiration pathways and the differentially regulated genes in those pathways are displayed using the MapMan tool. Each differentially regulated gene is displayed as a colored square. Fold changes of their transcript accumulation in each germination process were log3 transformed and displayed as a color scale from red (down-regulated) to blue (up-regulated) in the squares. The members of the same gene family are grouped and displayed together. Differentially regulated genes in early germination (left, top), late germination (right, top) and post-germination are displayed separately.

In the Photosynthesis pathway, post-germination up-regulated genes are over-represented in the sub-bins of the Light reaction pathways, the Photosystems I and II, Photorespiration and Rubisco Small Subunit Family (Figure 8). Figure 9 shows the differentially regulated genes in Light Reactions, Calvin Cycle and Photorespiration pathways in all three germination phases. A total of thirty-five genes were differentially regulated in the post-germination phase. Thirty-three of the 35 genes were up-regulated in the post-germination phase. In contrast, early and late germination up-regulated genes and early germination down-regulated genes were under-represented in the Photosynthesis pathways. Thus, the expression of the genes in the photosynthesis pathways changed little in the other germination phases, and the photosynthesis pathways were specifically up-regulated in the post-germination phase (Figure 8). Interestingly, genes encoding chloroplast/mito ribosomal proteins in protein synthesis pathways were also over-represented in post-germination up-regulated genes and under-represented in early and late germination up-regulated genes (Figure 8). Thus, the photosynthesis pathways and chloroplast/mito protein synthesis machinery in chloroplast were specifically and coordinately up-regulated in the post-germination phase and are likely to support the transition of seed germination from heterotrophic growth to photo-autotrophic growth. All the samples examined in the studies were germinated in dark. The specific up-regulation of photosynthesis pathways and chloroplast/mito ribosomal proteins over post-germination phase suggests that plants already develop a machinery or potential in the post-germination phase for light response and photosynthesis even in the dark to support their autotrophic growth.

In addition, post-germination up-regulated genes were over-represented in many degradation pathways and their related transfer protein families. Those include Starch Degradation and Starch Cleavage in Major CHO metabolism, lipid transfer proteins, beta-oxidation in lipid degradation, a variety of amino acid degradation pathways, nucleotide degradation, gluco-, galacto -and mannosidases, beta 1,3 glucan hydrolyases, nitrilase, acid and other phosphatases, GDSL_motif lipase and ribonucleases. It is consistent to patterns of amylase activity over course of seed germination. Up-regulation of individual or limited number of hydrolytic enzyme activities and genes during seed germination were documented in many publications [24]. However, the over-representation analysis of post-germination regulated genes provides strong evidence that those seed storage mobilization pathways were preferentially up-regulated during post-germination at a system level. Interestingly, no protein degradation pathway was up-regulated over the post-germination phase. Serine Proteases in protein degradation pathways were preferentially down-regulated while sucrose syntheses, glutamine synthase in N-Metabolism and central amino acid synthesis were up-regulated in post-germination phase. Thus, those post-germination up-regulated pathways and gene families may provide additional amino acid and sucrose resource for seedling growth.

Post-germination down-regulated genes were over-represented in the bins and sub-bins of Storage proteins, LEA and inhibitor proteins, unspecified seed proteins, serine protease, raffinose synthesis family, HRGP cell wall proteins, TAG synthesis in lipid metabolism, protein synthesis, mitochondria targeting pathways, and stress. Some of these, such as LEA and stress related functionalities were also preferentially down-regulated in late germination. Some of the functional categories, such as LEA, TAG synthesis and stresses-related bins were highly expressed during seed development and maturation. It is likely that the mRNA species in the pathways are degraded due to decreased needs for their functionalities in seed germination, which is consistent with the observation that some seed storage protein transcripts are degraded over the course of germination [31].

The representation analysis clearly shows that the transcriptional program underlying post-germination activates various mobilization pathways to degrade the storage reserve accumulated in mature grains and to meet increasing demands of vigorous seedling growth for energy and nutrients. Meanwhile, the growing seedlings also begin to turn on the transcriptional expression of photosynthesis pathways to switch from heterotrophic growth to photoautotrophic growth. Furthermore, transcriptional expression of soybean storage proteins, TAG synthesis and stress tolerance, which are highly expressed over the seed development and maturation, were suppressed over the post-germination phase to conserve the energy and nutrient for seedling growth.

#### Differential Expression Patterns of Biological Pathways

Interestingly, the up-regulated functionalities were over-represented in only one of three germination phases. None of them were preferentially up-regulated in more than two germination phases, suggesting that each germination phase transcriptionally induces a distinct set of biological functionalities over the course of germination. However, several functional categories related to stresses, development and LEA were down regulated over more than two phases. Most of the mRNA species in those functional categories were synthesized over the seed maturation and often highly accumulated in mature grains. They are likely to be degraded progressively in a less strictly regulated manner over the course of seed germination. However, no bin or sub-bin was over-represented in both up- regulated genes and down-regulated genes in the same germination phase. However, a number of functional categories were over-represented in up (down)-regulated genes, but under-represented in down (up)-regulated genes in the same germination phase. This suggests that plants have evolved a sophisticated and well regulated transcriptional mechanism to suppress antagonistic regulation of the same functional categories in the same germination phase to efficiently use the energy to support seed germination and seedling growth.

### Transcriptional Regulatory Programs Underlying GA and ABA Regulation of Seed Germination

**Figure 10:**
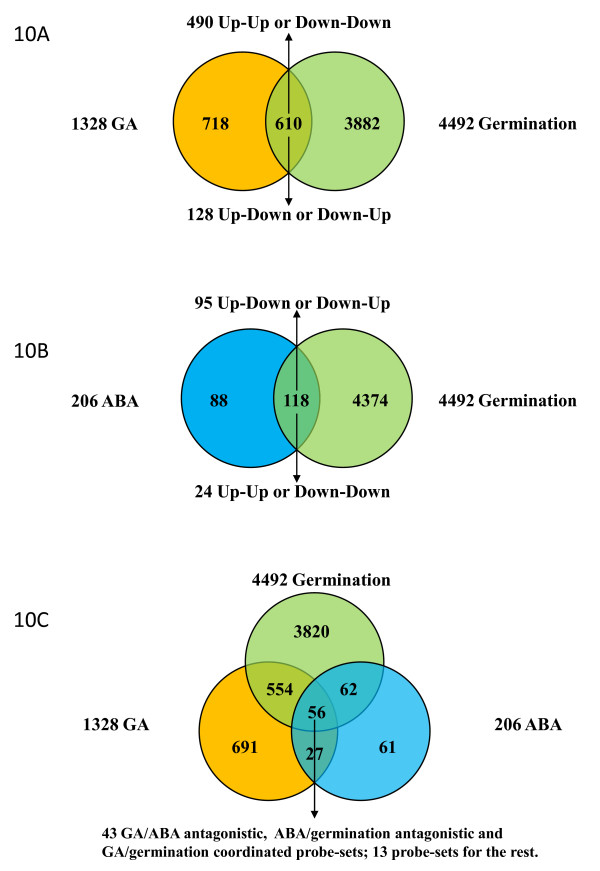
**Venn Diagram of the Genes Differentially Regulated by Germination, GA or ABA**. Germination, GA and ABA differentially regulated genes were compared and displayed as Venn diagrams. 10A compares GA and germination; 10B compares ABA and germination; and 10C shows the comparison of GA, ABA and germination. The number of probe-sets and their expression patterns are shown.

We previously examined transcriptomes of isolated germinating barley aleurone treated with GA and ABA respectively, and identified 1328 GA-responsive genes and 206 ABA-responsive genes in their mRNA accumulation [20]. Comparing the GA or ABA responsive genes with 4493 germination responsive genes showed that 46% of the GA responsive genes and 57% of the ABA responsive genes were also differentially regulated during germination (Figure 10A and 10B), suggesting that a large portion of GA or ABA responsive genes identified in isolated aleurone cells are likely involved in seed germination.

A total of 610 genes were differentially regulated by both the GA treatment and germination. Of the 610 genes, 490 (80%) genes showed a coordinate response to GA and germination (See Additional file 5), while only 128 genes showed an antagonistic response to GA treatment and germination (Figure 10A). The number of coordinately regulated genes was 3.8 times that of antagonistic regulated genes. The preferentially coordinate response of the genes to GA and germination provides strong evidence at a systems level for the hypothesis that GA enhances seed germination and seedling growth [4,5,12]. The coordinately regulated genes are potentially involved in GA regulation of germination and seedling growth processes.

**Figure 11:**
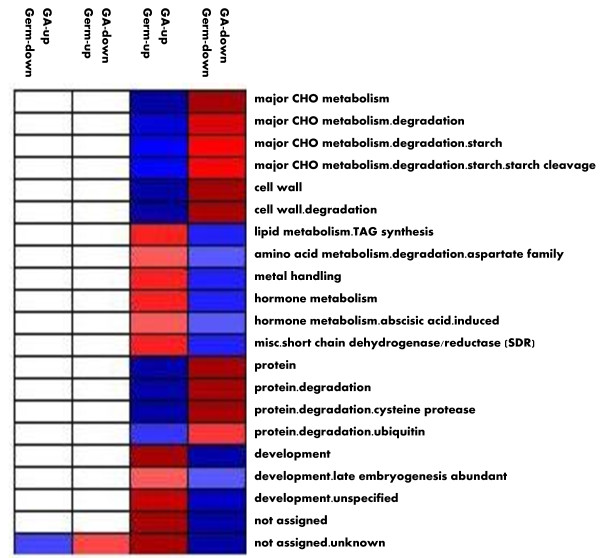
**Over-Representation of the Genes Differentially Regulated by Both GA and Germination**. The representation analysis was conducted on the 490 genes coordinately regulated by both GA and germination. Fold changes in their transcript accumulation in response to GA treatment was used in this analysis. Fisher's exact test and ORA Cutoff value of 1 were used. A false color scale of 3 indicates the statistic Z value. Blue indicates significance in over-representation while red denotes the significance in under-representation. Over- and under-represented bins are displayed using PageMan Tool and annotated with functional categories on the right and the expression patterns on the top.

Representation analysis revealed that the major CHO, cell wall and protein degradation pathways, which also included starch degradation, starch cleavage, cell wall degradation, cysteine protease and ubiquitin degradation pathways, were preferentially up-regulated by both GA and germination. However, TAG synthesis, Aspartate family degradation, metal handling, Hormone metabolism, ABA induced genes, Short chain dehydrogenase/reductase (SDR), and Late embryogenesis abundant genes were preferentially down-regulated by both GA and germination. Thus, it is likely that GA enhances seed germination and seedling growth partly through inducing mobilization of cell wall, starch and protein, and suppressing production of many seed maturation proteins such as LEA, TAG synthesis and ABA activated functions. Interestingly, the studies did not observed that the genes antagonistically regulated by GA and germination were over-represented significantly in any functional bins or sub-bins (Figure 11).

Out of 206 ABA responsive genes, 118 were also differentially regulated by germination. 95 of the 118 genes (80%) showed an antagonistic response to ABA treatment and germination (Figure 10B). Sixty-four genes were up-regulated by ABA but down-regulated by germination. Many of them encode embryogenesis abundant proteins and stress-related proteins. For example, 11 late embryogenesis abundant protein genes, 3 dehydrin genes (Contig1721_at, Contig1718_s_at, Contig1709_at) and three Glutathione-S-transferase genes (Contig14304_at, Contig2248_at, Contig2975_s_at) (See Additional file 6). The genes down-regulated by ABA and up-regulated by germination included alpha amylase, 3 beta-glucanase, beta-xylanase and cysteine proteinases. It has been well established that ABA promote establishing and maintaining seed dormancy, and inhibiting seed germination [13,18]. The studies provide systems evidences supporting that ABA suppresses germination partly through inhibiting storage reserve mobilization and enhancing expression of maturation genes.

A total of 56 germination responsive genes were differentially regulated by GA and ABA (See Figure 10C and See Additional file 7). Forty-three out of the 56 germination responsive genes were regulated coordinately by GA, and antagonistically by ABA. Seventeen of the genes were up-regulated by both GA and germination, but down-regulated by ABA. Twenty six genes were down-regulated by both GA and germination, but up-regulated by ABA. Many of the genes encoded embryogenesis abundant and stress-related proteins, which include 10 LEA proteins, one GST and one dehydrin. Four regulatory genes encoding two protein phosphatase 2C, a WRKY transcription factor and an AP2-domain DNA-binding protein were identified in the set of genes. It has been proposed that GA and ABA play antagonistic roles in regulating seed maturation and germination [4,5]. Those genes are likely to be important part of the pathway mediating the antagonistic interaction of GA and ABA signaling pathways in regulating seed germination. The four transcription factor and signaling genes may play a regulatory role in the networks. It should be noted that those GA and ABA responsive genes are identified in barley aleurone. However, GA and ABA regulation of seed germination and seedling growth could occur at multiple tissues including embryo tissues. It remains to be determined if those genes are differentially regulated by GA and ABA in those tissues as in barley aleurone.

## Conclusions

The studies carefully examined water content, loss of desiccation tolerance, amylase activity and morphology of germinating barley, and selected six distinct developmental stages for transcriptome analysis based on the multi-germination characteristics and relative timing of germination. The studies developed a model depicting transcriptional regulatory program underlying barley germination at gene, pathway and systems levels. Prior to grain imbibition, mature barley grains already accumulate a large number of transcripts, which are synthesized during seed development and maturation and preserved in mature grains. Although accumulation of some of those transcripts decreases over the course of barley germination, a significant number of the transcripts are likely to remain in germinating barley and participate in seed germination and seedling growth. Comparing highly abundant transcripts in barley and *Arabidopsis *dry seeds showed that those barley and *Arabidopsis *transcripts in dry seeds are highly conserved, and suggested the ancient origins of those highly abundant seed transcripts and their functional significance in germination. Upon grain imbibition, a new transcriptional regulatory program is activated quickly, which could occur as early as within the first three hours of imbibition. Hierarchal clustering of the transcriptomes of germinating barley at each developmental stage reveals that the new transcriptional regulatory program is composed of three distinct phases, early, late, and post-germination phases. Early germination phase represents the first nine-hour germination and preferentially induces genes encoding regulatory components including transcription factors, signaling components and post-translational modification proteins. Those regulatory genes are likely to activate a variety of transcriptional regulatory cascades and signaling transduction pathways in seed germination and seedling growth. In addition, cell wall synthesis and modification pathway genes are also preferentially up-regulated within the early germination phase, which may function to loosen cell walls for subsequent cell expansion and division, and radicle protrusion. Within the following 9 hours of late germination phase, genes encoding many metabolic pathway enzymes and cellular components including amino acid and nucleotide synthesis, protein degradation, chromatin remodeling and cell division pathways are preferentially up-regulated to provide nutrient and cellular components for cell division and elongation. In addition, a transcriptional switch occurs in the late germination phase, and correlates with the developmental transition from germination to seedling growth. Post-germination phase mainly represents seedling growth process after coleorhiza emergence from the germinating grains. As expected, seed reserve mobilization and photosynthesis pathway genes are preferentially induced in the phase to mobilize seed storage reserve in mature grains, and acquire autotrophic growth ability to meet the increasing demand for energy and nutrients in seedling growth. In addition, transcriptional expression of secondary metabolism and tetrapyrrole synthesis pathway genes are up-regulated during post-germination phase. However, many genes encoding stress related protein, LEA genes and seed storage proteins, which are highly expressed during seed development and maturation, are transcriptionally suppressed over the course of barley germination to conserve the energy and nutrients for seed germination and seedling growth. Although some of those metabolic pathways have been previously proposed or logically assumed to play roles in germination, the studies illustrated that transcriptional expression of those pathways are differentially regulated over the course of germination at a systems level, and provided additional evidences for their roles in germination. The studies also newly discovered a number of pathways and genes that are differentially regulated over the course of germination at a transcriptional level, and suggested their functional involvement in germination. A great number of hypotheses also have been developed in the studies for future validation. In addition, the studies identified a set of genes encoding regulatory components that were transiently up-regulated as early as 3 hours of imbibition. Their transient and up-regulated expression patterns at such an early stage of germination suggests that they may play key regulatory functions in seed germination, and worth further investigating their functions in seed germination.

The studies also compared GA and ABA responsive genes with genes differentially regulated by barley germination, and identified three sets of germination responsive genes that coordinately respond to GA, antagonistically respond to ABA, and coordinately respond to GA but antagonistically respond to ABA. Those genes are likely to be important components in the transcriptional regulatory networks that GA enhances germination, ABA suppresses germination, and GA and ABA interact antagonistically in regulating germination.

Overall, the studies establish a standard transcriptome reference platform for barley germination and enable seed biologists to integrate the transcriptome data with a variety of -omics and other biological data to illustrate biological networks underlying barley germination. The studies also developed a model depicting the transcriptional regulatory programs underlying seed germination and germination related biological pathways, and GA and ABA regulation of seed germination at gene, pathway and systems levels.

## Methods

### Plant Growth and Harvest

Plump and healthy barley grains (*Hordeum vulgare *L. cv. Morex L.) were imbibed in water for three hours at 22°C with three changes of water and were then germinated on water-saturated germination pack in the dark at 22°C. Twenty grains were planted in each 15 cm diameter Petri-dish and spaced evenly to reduce the variation of seed germination caused by grain density. Over 98% of grains germinated at these conditions, but individual grains did not germinate uniformly. The germinating grains/developing seedlings with typical morphologies at 0 (dry), 3, 9, 18, 33, and 71 hours of germination were harvested and pooled for determination of water content, alpha amylase activity, loss of desiccation resistance and RNA purification. The typical morphology of the seedlings at 18, 33, and 71 hours of germination was identical to that of S3, S4 and S5 stages, (Figure 1A). The grains/seedlings harvested for RNA extraction and determination of alpha amylase activity were pooled together and immediately frozen in liquid nitrogen and stored at -80°C. Each replication of water content, amylase activity, desiccation resistance and microarray assays represented an independent germination experiment. Three independent germination experiments were conducted for each time point. Each replication represents a pool of germinating barleys that were carefully selected from an independent germination experiment based on the relative time point and morphology defined for the given stage to reduce the heterogeneity of the pooled barley tissues potentially caused by their different germination rate.

### Alpha-Amylase Assay

Alpha amylase activity was measured using a DNSA assay [70]. One-half gram of tissue was homogenized using a Bead-Beater at maximum speed for 2 min in 500 ml of phosphate buffer (20 mM Na_2_HPO_4_, 10 mM NaCl, pH 6.9). The sample was then centrifuged for 10 min at 13,000 rpm. The aqueous phase was incubated at 69°C for 15 min to inactivate beta-amylase. After centrifugation for 10 min at 13,000 rpm, 10 mL of solution was added to a phosphate buffer with 0.5% starch and incubated at 30°C for 30 min. Then, an equal amount of DNSA reagent (0.25 mM NaO, 1% 3,5-dinitrosalicylic acid, 30% NaK tartrate) was added to the reaction and incubated for 15 min at 100°C. After cooling the sample to 22°C and centrifuging for 5 min at 13,000 rpm, 200 ml of supernatant was added to microplates to measure the OD at the wavelength of 547 nm. Maltose was used as the standard to calculate enzyme activity. Dry grains were used as a control for amylase activity.

### Desiccation Resistance Assay

For the desiccation resistance assay, the grains/seedlings with the representative morphology at each given stage (Figure 1A) were harvested and dried aerially at room temperature for two weeks. The dehydrated grains/seedlings were re-germinated at the same condition as described in the germination experiment. The desiccation resistance of the grains at each stage was determined as percentage of survival, defined as the percentage of the dehydrated grains/seedlings that can revive within three days of germination. Sample size for each desiccation resistance assay ranged from 23 grains to 56 grains. Three independent replications were conducted for each time point.

### RNA Purification

Total RNA extraction was conducted as described by Chen and An with minor modifications [20]. Two grams of plant tissue was ground in liquid nitrogen followed by the addition of 10 ml extract buffer (4% *p*-aminosalicylic disodium, 1% 1,5-napthalenedisulfonic acid) and 10 ml phenol. The mixture was inverted several times, and polytroned for 45 seconds after the addition of 10 ml chloroform. After centrifuging, the aqueous phase was transferred into a new tube, to which 60 ml of 10% Calcoflur White was added [70]. The mixture was mixed thoroughly and centrifuged for 15 min at 4°C, 12,000 rpm. Precipitate RNA from the supernatant was formed by using 1/10 volume of 3M NaOAc, and 2 volume of 100% ethanol. After centrifuging, the RNA pellet was first dissolved in 8 ml water, then 5 ml of 8M LiCl was added, and the tubes were left on ice overnight. After centrifuging, the resulting RNA pellet was dissolved in water. The RNA quality and quantity was checked by using Nano-Drop (Nano-Drop, Wilmington, DE) and Aglient 2100 Bioanalyzer (Aglient, Palo Alto, CA).

### GeneChip Array Assay

Preparations of cDNA and biotin-labeled cRNA were performed and analyzed as recommended by Affymetrix, Inc. (Santa Clara, CA). According to the manufacturer's protocol, 7.5 to 15 mg of total RNA was used in a reverse transcription reaction to generate first-strand cDNA using Reverse transcriptase SuperScript II (Invitrogen, Carlsbad, CA). After second-strand synthesis, double-strand cDNAs were used in an *in vitro *transcription reaction to generate biotinylated cRNA. Ten mg of fragmented cRNA was used for each hybridization. Staining and scanning of the hybridized GeneChips were performed as described by Chen and An [20]and the manufacturer's recommended protocols (Affymetrix, Inc., Santa Clara, CA). The .CEL files from all samples are available in GEO (http://www.ncbi.nlm.nih.gov/geo/info/linking.html) with series record number of GSE23595 and access numbers of GSM578686 to GSM578703.

### Data acquisition and analysis

Affymetrix GeneChip Microarray Suite version 5.0 software (MAS 5.0) was used to assign the presence and absence calls of each probe set for each GeneChip with a P value of 0.05. The data files containing the probe set intensities (.cel files) were used for background correction and normalization by an improved log2 scale RMA procedure, GC-RMA, provided in GeneSpring Suite 7.2. Within each array, a further "per gene normalize the median" (with cutoff 0.01) was applied to the pre-normalized data using GC-RMA provided in the GeneSpring 7.2 software. The probe sets with absence calls across all chips were removed from further analysis. A probe set with present (or absent) calls in two of the three replicates were assigned as present (or absence) call for the treatment. We used the GC-RMA approach to convert probe level data to expression measurement in the microarray experiments. Compared with the algorithms used in Microarray Suites (MAS 5.0), this approach adjusts background on the raw intensity value scale, then uses quartile normalization to remove systemic variations, and summarizes log2 of the normalized background adjusted PM values to estimate expression level measurements based on a linear additive model [39]. One-way ANOVA was used to identify genes that were differentially expressed at any two time points during seed germination with a False Discovery Rate (FDR) of 0.05. The Parametric Test, Variances Assumed Equal Option, Benjamini and Hochberg multiple testing corrections were used in the one-way ANOVA analysis. The harvEST:Barley (version 1.35; http://harvest.ucr.edu/), Munich Information Center for Protein Sequence [71] and Universal Protein Resource [72], were used to conduct gene functional annotation in addition to manual editing. An E score of 1 × E^-20 ^of BLAST-X between a barley sequence and *Arabidopsis *sequences in HarvEST: Barley was used as a cutoff.. The *Arabidopsis *genes homologous to a given barley gene with the lowest e-value were defined as putative *Arabidopsis *orthologs. MapMan (version 1.4.3) and PageMan (Version 0.12) were used for over-representation functional analysis [62,73].

## Authors' contributions

YQA conceived, designed and coordinated the studies. YQA participated in morphological and physiological characterization of barley germination and microarray assays. LL carried out most of microarray assays. YQA and LL performed bioinformatic data analysis and interpretation, and drafted the manuscript. All authors read and approved the final manuscript.

## Supplementary Material

Additional file 1 **Genes differentially regulated between any stages vs. S0 and between adjacent stages**. Click here for file

Additional file 2 **Summary of Differentially Regulated Genes over the Course of Barley Germination**. Click here for file

Additional file 3 **List of Probe-sets Differentially Regulated Based on Germination Phases**. Click here for file

Additional file 4 **Early Germination Regulated Cell Wall Genes**. Click here for file

Additional file 5 **Genes Differentially Regulated by Both GA and Germination Genes**. Click here for file

Additional file 6 **Genes Differentially Regulated by Both ABA and Germination**. Click here for file

Additional file 7 **Genes Differentially Regulated by GA, ABA and Germination**. Click here for file

## References

[B1] Bewley JD Seed Germination and Dormancy Plant Cell 1997 9 7 1055 1066 10.1105/tpc.9.7.1055 12237375 PMC156979

[B2] Bewley JD Black M Seeds: physiology of development and germination 1985 New York: Plenum Press

[B3] Mayer AM Shain Y Control of Seed Germination Annual Review of Plant Physiology 1974 25 1 167 193 10.1146/annurev.pp.25.060174.001123

[B4] White CN Proebsting WM Hedden P Rivin CJ Gibberellins and Seed Development in Maize. I. Evidence That Gibberellin/Abscisic Acid Balance Governs Germination versus Maturation Pathways Plant Physiol 2000 122 4 1081 1088 10.1104/pp.122.4.1081 10759503 PMC58942

[B5] White CN Rivin CJ Gibberellins and Seed Development in Maize. II. Gibberellin Synthesis Inhibition Enhances Abscisic Acid Signaling in Cultured Embryos Plant physiology 2000 122 4 1089 1098 10.1104/pp.122.4.1089 10759504 PMC58943

[B6] Yamaguchi S Smith MW Brown RGS Kamiya Y Sun Tp Phytochrome Regulation and Differential Expression of Gibberellin 3ß-Hydroxylase Genes in Germinating Arabidopsis Seeds Plant Cell 1998 10 12 2115 2126 9836749 10.1105/tpc.10.12.2115 PMC143973

[B7] Koornneef M Veen JH Induction and analysis of gibberellin sensitive mutants in Arabidopsis thaliana (L.) heynh TAG Theoretical and Applied Genetics 1980 58 6 257 263 10.1007/BF00265176 24301503

[B8] Liu Y Bergervoet JHW Vos CHR Hilhorst HWM Kraak HL Karssen CM Bino RJ Nuclear replication activities during imbibition of abscisic acid- and gibberellin-deficient tomato (Lycopersicon esculentum Mill.) seeds Planta 1994 194 3 368 373

[B9] Sun Tp Gubler F Molecular Mechanism of Gibberellin Signaling in Plants Annual Review of Plant Biology 2004 55 1 197 223 10.1146/annurev.arplant.55.031903.141753 15377219

[B10] Peng J Richards DE Hartley NM Murphy GP Devos KM Flintham JE Beales J Fish LJ Worland AJ Pelica F `Green revolution' genes encode mutant gibberellin response modulators Nature 1999 400 6741 256 261 10.1038/22307 10421366

[B11] McGinnis KM Thomas SG Soule JD Strader LC Zale JM Sun TP Steber CM The Arabidopsis SLEEPY1 gene encodes a putative F-box subunit of an SCF E3 ubiquitin ligase Plant Cell 2003 15 5 1120 1130 10.1105/tpc.010827 12724538 PMC153720

[B12] Peng J Harberd NP The role of GA-mediated signalling in the control of seed germination Curr Opin Plant Biol 2002 5 5 376 381 10.1016/S1369-5266(02)00279-0 12183174

[B13] Finkelstein R Reeves W Ariizumi T Steber C Molecular aspects of seed dormancy Annu Rev Plant Biol 2008 59 387 415 10.1146/annurev.arplant.59.032607.092740 18257711

[B14] Jacobsen JV Gibberellin action in germinated cereal grains 1995 Dordrecht: The Netherlands: Kluwer Academic Publishers

[B15] King RW Abscisic acid in developing wheat grains and its relationship to grain growth and maturation Planta 1976 132 43 51 10.1007/BF00390329 24424906

[B16] Black Mlnvolvement of ABA in the physiology of developing and maturing seeds 1991 Oxford, UK: BlOS Scientific

[B17] Finkelstein R Tenbarge K Shumway J Crouch M Role of abscisic acid in maturation of rape seed embryos Plant Physiol 1985 78 630 636 10.1104/pp.78.3.630 16664296 PMC1064789

[B18] Finkelstein RR Gampala SS Rock CD Abscisic acid signaling in seeds and seedlings Plant Cell 2002 14 Suppl S15 45 12045268 10.1105/tpc.010441 PMC151246

[B19] Hoecker U Vasil IK McCarty DR Signaling from the embryo conditions Vp1-mediated repression of alpha-amylase genes in the aleurone of developing maize seeds Plant J 1999 19 4 371 377 10.1046/j.1365-313X.1999.00521.x 10504559

[B20] Chen K An YQ Transcriptional Responses to Gibberellin and Abscisic Acid in Barley Aleurone Journal of Integrative Plant Biology 2006 48 5 591 612 10.1111/j.1744-7909.2006.00270.x

[B21] Belin C Megies C Hauserova E Lopez-Molina L Abscisic Acid Represses Growth of the Arabidopsis Embryonic Axis after Germination by Enhancing Auxin Signaling Plant Cell 2009 21 8 2253 2268 10.1105/tpc.109.067702 19666738 PMC2751952

[B22] Linkies A Muller K Morris K Tureckova V Wenk M Cadman CSC Corbineau F Strnad M Lynn JR Finch-Savage WE Ethylene Interacts with Abscisic Acid to Regulate Endosperm Rupture during Germination: A Comparative Approach Using Lepidium sativum and Arabidopsis thaliana Plant Cell 2009 21 12 3803 3822 10.1105/tpc.109.070201 20023197 PMC2814513

[B23] MacGregor AW Bhatty RS Barley: chemistry and technology.: Amer Assn of Cereal Chemists 1993

[B24] Fincher GB Molecular and Cellular Biology Associated with Endosperm Mobilization in Germinating Cereal Grains Annual Review of Plant Physiology and Plant Molecular Biology 1989 40 1 305 346 10.1146/annurev.pp.40.060189.001513

[B25] Chrispeels Mj Varner JE Gibberellic Acid-Enhacned Synthesis and Relesase of alpha Amylase and Ribonuclease by Isolated Barley Aleurone Layers Plant Physiol 1967 398 406 10.1104/pp.42.3.398 PMC1086548 16656517

[B26] Chen K Tian S Yandell B Kaeppler S An YQ Loss-of-function of DELLA protein SLN1 activates GA signaling in barley aleurone Acta Physiologiae Plantarum 2010

[B27] Ogawa M Hanada A Yamauchi Y Kuwahara A Kamiya Y Yamaguchi S Gibberellin Biosynthesis and Response during Arabidopsis Seed Germination Plant Cell 2003 15 7 1591 1604 10.1105/tpc.011650 12837949 PMC165403

[B28] Nakabayashi K Okamoto M Koshiba T Kamiya Y Nambara E Genome-wide profiling of stored mRNA in *Arabidopsis thaliana *seed germination: epigenetic and genetic regulation of transcription in seed The Plant Journal 2005 41 5 697 709 10.1111/j.1365-313X.2005.02337.x 15703057

[B29] Preston J Tatematsu K Kanno Y Hobo T Kimura M Jikumaru Y Yano R Kamiya Y Nambara E Temporal expression patterns of hormone metabolism genes during imbibition of Arabidopsis thaliana seeds: a comparative study on dormant and non-dormant accessions Plant Cell Physiol 2009 50 10 1786 1800 10.1093/pcp/pcp121 19713425

[B30] Holdsworth MJ Finch-Savage WE Grappin P Job D Post-genomics dissection of seed dormancy and germination Trends in Plant Science 2008 13 1 7 13 10.1016/j.tplants.2007.11.002 18160329

[B31] Potokina E Sreenivasulu N Altschmied L Michalek W Graner A Differential gene expression during seed germination in barley (Hordeum vulgare L.) Funct Integr Genomics 2002 2 1-2 28 39 10.1007/s10142-002-0050-x 12021848

[B32] White J Pacey-Miller T Crawford A Cordeiro G Barbary D Bundock P Henry R Abundant transcripts of malting barley identified by serial analysis of gene expression (SAGE) Plant Biotechnol J 2006 4 3 289 301 10.1111/j.1467-7652.2006.00181.x 17147635

[B33] Sreenivasulu N Usadel B Winter A Radchuk V Scholz U Stein N Weschke W Strickert M Close TJ Stitt M Barley grain maturation and germination: metabolic pathway and regulatory network commonalities and differences highlighted by new MapMan/PageMan profiling tools Plant Physiol 2008 146 4 1738 1758 10.1104/pp.107.111781 18281415 PMC2287347

[B34] Barrero JM Talbot MJ White RG Jacobsen JV Gubler F Anatomical and Transcriptomic Studies of the Coleorhiza Reveal the Importance of This Tissue in Regulating Dormancy in Barley Plant Physiol 2009 150 2 1006 1021 10.1104/pp.109.137901 19386806 PMC2689963

[B35] Watson L Henry RJ Microarray analysis of gene expression in germinating barley embryos (&lt;i&gt;Hordeum vulgare&lt;/i&gt; L.) Functional &amp; Integrative Genomics 2005 5 3 155 162 10.1007/s10142-005-0133-6 15714320

[B36] Close TJ Wanamaker SI Caldo RA Turner SM Ashlock DA Dickerson JA Wing RA Muehlbauer GJ Kleinhofs A Wise RP A New Resource for Cereal Genomics: 22K Barley GeneChip Comes of Age Plant Physiol 2004 134 3 960 968 10.1104/pp.103.034462 15020760 PMC389919

[B37] Rajjou L Gallardo K Debeaujon I Vandekerckhove J Job C Job D The effect of alpha-amanitin on the Arabidopsis seed proteome highlights the distinct roles of stored and neosynthesized mRNAs during germination Plant Physiol 2004 134 4 1598 1613 10.1104/pp.103.036293 15047896 PMC419834

[B38] Kimura M Nambara E Stored and neosynthesized mRNA in Arabidopsis seeds: effects of cycloheximide and controlled deterioration treatment on the resumption of transcription during imbibition Plant Molecular Biology 2010 73 1 119 129 10.1007/s11103-010-9603-x 20099072

[B39] Irizarry RA Bolstad BM Collin F Cope LM Hobbs B Speed TP Summaries of Affymetrix GeneChip probe level data Nucl Acids Res 2003 31 4 e15 10.1093/nar/gng015 12582260 PMC150247

[B40] Gallardo K Job C Groot SPC Puype M Demol H Vandekerckhove J Job D Proteomic Analysis of Arabidopsis Seed Germination and Priming Plant Physiol 2001 126 2 835 848 10.1104/pp.126.2.835 11402211 PMC111173

[B41] Buchanan BB Balmer Y REDOX REGULATION: A Broadening Horizon Annual Review of Plant Biology 2005 56 1 187 220 10.1146/annurev.arplant.56.032604.144246 15862094

[B42] Yano H Wong JH Cho MJ Buchanan BB Redox Changes Accompanying the Degradation of Seed Storage Proteins in Germinating Rice Plant and Cell Physiology 2001 42 8 879 883 10.1093/pcp/pce119 11522916

[B43] Wolfe KH Gouy M Yang YW Sharp PM Li WH Date of the monocot-dicot divergence estimated from chloroplast DNA sequence data Proceedings of the National Academy of Sciences of the United States of America 1989 86 16 6201 6205 10.1073/pnas.86.16.6201 2762323 PMC297805

[B44] Li WH Yang J Gu X Expression divergence between duplicate genes Trends in Genetics 2005 21 11 602 607 10.1016/j.tig.2005.08.006 16140417

[B45] An YQ Huang S McDowell JM McKinney EC Meagher RB Conserved expression of the Arabidopsis ACT1 and ACT 3 actin subclass in organ primordia and mature pollen Plant Cell 1996 8 1 15 30 8597657 10.1105/tpc.8.1.15 PMC161078

[B46] An YQ McDowell JM Huang S McKinney EC Chambliss S Meagher RB Strong, constitutive expression of the Arabidopsis ACT2/ACT8 actin subclass in vegetative tissues Plant J 1996 10 1 107 121 10.1046/j.1365-313X.1996.10010107.x 8758981

[B47] Emerson JJ Li WH The genetic basis of evolutionary change in gene expression levels Philosophical Transactions of the Royal Society B: Biological Sciences 2010 365 1552 2581 2590 10.1098/rstb.2010.0005 PMC2935095 20643748

[B48] Graham IA Seed Storage Oil Mobilization Annual Review of Plant Biology 2008 59 1 115 142 10.1146/annurev.arplant.59.032607.092938 18444898

[B49] Huang AH Oleosins and oil bodies in seeds and other organs Plant Physiol 1996 110 4 1055 1061 10.1104/pp.110.4.1055 8934621 PMC160879

[B50] Siloto RMP Findlay K Lopez-Villalobos A Yeung EC Nykiforuk CL Moloney MM The Accumulation of Oleosins Determines the Size of Seed Oilbodies in Arabidopsis Plant Cell 2006 18 8 1961 1974 10.1105/tpc.106.041269 16877495 PMC1533971

[B51] Jolivet P Roux E d'Andrea S Davanture M Negroni L Zivy M Chardot T Protein composition of oil bodies in Arabidopsis thaliana ecotype WS Plant Physiology and Biochemistry 2004 42 6 501 509 10.1016/j.plaphy.2004.04.006 15246063

[B52] Katavic V Agrawal GK Hajduch M Harris SL Thelen JJ Protein and lipid composition analysis of oil bodies from two Brassica napus cultivars Proteomics 2006 6 16 4586 4598 10.1002/pmic.200600020 16847873

[B53] Aalen RB The transcripts encoding two oleosin isoforms are both present in the aleurone and in the embryo of barley (Hordeum vulgare L.) seeds Plant Molecular Biology 1995 28 3 583 588 10.1007/BF00020404 7632926

[B54] Zentella R Zhang ZL Park M Thomas SG Endo A Murase K Fleet CM Jikumaru Y Nambara E Kamiya Y Global Analysis of DELLA Direct Targets in Early Gibberellin Signaling in Arabidopsis Plant Cell 2007 10.1105/tpc.107.054999 PMC2174696 17933900

[B55] Howell KA Narsai R Carroll A Ivanova A Lohse M Usadel B Millar AH Whelan J Mapping Metabolic and Transcript Temporal Switches during Germination in Rice Highlights Specific Transcription Factors and the Role of RNA Instability in the Germination Process Plant Physiol 2009 149 2 961 980 19074628 10.1104/pp.108.129874 PMC2633829

[B56] Hirayama T Shinozaki K Perception and transduction of abscisic acid signals: keys to the function of the versatile plant hormone ABA Trends in Plant Science 2007 12 8 343 351 10.1016/j.tplants.2007.06.013 17629540

[B57] Gosti F Beaudoin N Serizet C Webb AAR Vartanian N Giraudat J ABI1 Protein Phosphatase 2C Is a Negative Regulator of Abscisic Acid Signaling Plant Cell 1999 11 10 1897 1910 10521520 10.1105/tpc.11.10.1897 PMC144098

[B58] Xie Z Zhang ZL Zou X Huang J Ruas P Thompson D Shen QJ Annotations and Functional Analyses of the Rice WRKY Gene Superfamily Reveal Positive and Negative Regulators of Abscisic Acid Signaling in Aleurone Cells Plant Physiol 2005 137 1 176 189 10.1104/pp.104.054312 15618416 PMC548849

[B59] Holdsworth MJ Bentsink L Soppe WJJ Molecular networks regulating Arabidopsis seed maturation, after-ripening, dormancy and germination New Phytologist 2008 179 1 33 54 10.1111/j.1469-8137.2008.02437.x 18422904

[B60] Nambara E Okamoto M Tatematsu K Yano R Seo M Kamiya Y Abscisic acid and the control of seed dormancy and germination Seed Science Research 2010 20 02 55 67 10.1017/S0960258510000012

[B61] Usadel B Nagel A Thimm O Redestig H Blaesing OE Palacios-Rojas N Selbig J Hannemann J Piques MC Steinhauser D Extension of the Visualization Tool MapMan to Allow Statistical Analysis of Arrays, Display of Coresponding Genes, and Comparison with Known Responses Plant Physiol 2005 138 3 1195 1204 10.1104/pp.105.060459 16009995 PMC1176394

[B62] Usadel B Nagel A Steinhauser D Gibon Y Blasing OE Redestig H Sreenivasulu N Krall L Hannah MA Poree F PageMan: an interactive ontology tool to generate, display, and annotate overview graphs for profiling experiments BMC Bioinformatics 2006 7 535 10.1186/1471-2105-7-535 17176458 PMC1766370

[B63] Kucera B Cohn MA Leubner-Metzger G Plant hormone interactions during seed dormancy release and germination Seed Science Research 2005 15 04 281 307 10.1079/SSR2005218

[B64] Liu PP Koizuka N Martin RC Nonogaki H The BME3 (Blue Micropylar End 3) GATA zinc finger transcription factor is a positive regulator of Arabidopsis seed germination The Plant Journal 2005 44 6 960 971 10.1111/j.1365-313X.2005.02588.x 16359389

[B65] Tanaka M Kikuchi A Kamada H The Arabidopsis Histone Deacetylases HDA6 and HDA19 Contribute to the Repression of Embryonic Properties after Germination Plant physiology 2008 146 1 149 161 18024558 10.1104/pp.107.111674 PMC2230551

[B66] Liu Y Koornneef M Soppe WJJ The Absence of Histone H2B Monoubiquitination in the Arabidopsis hub1 (rdo4) Mutant Reveals a Role for Chromatin Remodeling in Seed Dormancy The Plant Cell Online 2007 19 2 433 444 10.1105/tpc.106.049221 PMC1867323 17329563

[B67] Tai H Tai G Beardmore T Dynamic Histone Acetylation of Late Embryonic Genes during Seed Germination Plant Molecular Biology 2005 59 6 909 925 10.1007/s11103-005-2081-x 16307366

[B68] Gu Z Nicolae D Lu HH-S Li WH Rapid divergence in expression between duplicate genes inferred from microarray data Trends in Genetics 2002 18 12 609 613 10.1016/S0168-9525(02)02837-8 12446139

[B69] Tunnacliffe A Wise M The continuing conundrum of the LEA proteins Naturwissenschaften 2007 94 10 791 812 10.1007/s00114-007-0254-y 17479232

[B70] Skadsen RW Aleurones from a Barley with Low [alpha]-Amylase Activity Become Highly Responsive to Gibberellin When Detached from the Starchy Endosperm Plant Physiol 1993 102 1 195 203 12231810 10.1104/pp.102.1.195 PMC158763

[B71] Schoof H Ernst R Nazarov V Pfeifer L Mewes HW Mayer KF MIPS Arabidopsis thaliana Database (MAtDB): an integrated biological knowledge resource for plant genomics Nucleic Acids Res 2004 32 Database issue D373 376 14681437 10.1093/nar/gkh068 PMC308802

[B72] Bairoch A Apweiler R Wu CH Barker WC Boeckmann B Ferro S Gasteiger E Huang H Lopez R Magrane M The Universal Protein Resource (UniProt) Nucleic Acids Res 2005 33 Database issue D154 159 15608167 10.1093/nar/gki070 PMC540024

[B73] Thimm O Bläsing O Gibon Y Nagel A Meyer S Krüger P Selbig J Müller LA Rhee SY Stitt M Mapman: a user-driven tool to display genomics data sets onto diagrams of metabolic pathways and other biological processes Plant Journal 2004 37 6 914 939 10.1111/j.1365-313X.2004.02016.x 14996223

